# Host Genetic Susceptibility and Antiviral Immunity Shape the Pathogenesis and Outcomes of Herpes Simplex Encephalitis

**DOI:** 10.3390/biology15181648

**Published:** 2026-09-18

**Authors:** Yufei Zhan, Jing Li, Qiaoling Yuan, Xianzheng Yin, Jie Tong

**Affiliations:** College of Life Sciences, School of Life Sciences and Green Development, Hebei University, Baoding 071002, China; zhan07133626@163.com (Y.Z.); lj011026@126.com (J.L.); luckhbu@163.com (Q.Y.); yxz2002@126.com (X.Y.)

**Keywords:** herpes simplex encephalitis (HSE), herpes simplex virus (HSV), Toll-like receptor 3 (TLR3), interferon (IFN), central nervous system (CNS) immunity

## Abstract

Herpes simplex encephalitis (HSE), predominantly caused by herpes simplex virus type 1 (HSV-1), is the most common sporadic viral encephalitis worldwide and remains life threatening despite antiviral therapy. Although herpes simplex virus (HSV) infection is highly prevalent, only a minority of individuals develop HSE, highlighting the critical contribution of host-specific factors to disease susceptibility. This review summarizes current knowledge regarding HSE epidemiology, antiviral immunity, genetic susceptibility, and therapeutic advances. Underlying inborn errors of immunity fall into three mechanistic groups: impaired innate immune sensing pathways, including Toll-like receptor 3 (*TLR3*), retinoic-acid-inducible gene-I (*RIG-I*)/melanoma differentiation-associated gene 5 (*MDA5*), and cyclic GMP-AMP synthase (*cGAS*)–stimulator of interferon genes (STING), which compromise type I interferon (IFN) production in central nervous system (CNS) resident cells; disrupted neuron intrinsic antiviral mechanisms represented by mutations in specific genes (e.g., *DBR1*, *SNORA31*, and *GTF3A*); and defects in other IFN signaling molecules, including interferon-α/β receptor 1 (*IFNAR1*) and 2 (*IFNAR2*), as well as signal transducer and activator of transcription 1 (*STAT1*) and 2 (*STAT2*), which further weaken antiviral defenses. These findings underscore cell autonomous antiviral responses within the brain and provide insights into the selective vulnerability to HSE. Emerging therapeutic strategies aim to integrate antiviral treatment with modulation of host immune responses and, potentially, personalized approaches based on genetic profiling. In all, we emphasize the pivotal role of CNS-intrinsic immunity and host genetic factors in shaping disease outcomes. A deeper understanding of the interplay between HSV and host defense mechanisms will facilitate the development of improved diagnostic tools and targeted therapies, ultimately reducing the burden of this devastating disease.

## Simple Summary

Herpes simplex encephalitis (HSE) is the most common sporadic viral encephalitis worldwide. Even with antiviral therapy, it remains life-threatening and often leaves survivors with severe neurological deficits. Although herpes simplex virus (HSV) infection is extremely common, only a tiny fraction of infected individuals develops HSE, pointing to host factors as key drivers of disease. Recent research has shown that intrinsic antiviral immunity within the central nervous system is critical. Inborn errors affecting the innate immune pathways and downstream molecules (e.g., UNC93B1, TRIF, TRAF3, TBK1, IRF3) impair type I interferon (IFN) production in brain-resident cells, allowing uncontrolled viral replication. Mutations in immune signaling components, such as IFNAR1, IFNAR2, STAT1, and STAT2, further weaken defenses. These genetic insights explain why certain people are vulnerable and open new therapeutic avenues. Future management should combine antiviral drugs with immune-modulating strategies and, potentially, genotype-guided personalized approaches. A clearer understanding of host–virus interactions will improve diagnostics and targeted treatments, ultimately reducing the heavy burden of this devastating disease.

## 1. Introduction

Herpes simplex virus (HSV) is a human pathogen that establishes lifelong latent infection following primary exposure [1]. HSV comprises two serotypes, HSV-1 and HSV-2, with estimated global seroprevalence rates of 67% and 13%, respectively [2]. Although HSV infection is typically asymptomatic or results in mild mucocutaneous disease, it can lead to severe complications under certain conditions, particularly in immunocompromised individuals or those with specific genetic susceptibilities [3,4].

Among these complications, herpes simplex encephalitis (HSE) represents the most severe manifestation of HSV infection in the central nervous system (CNS) [1]. HSE is characterized by acute inflammation of the brain parenchyma, most commonly affecting the temporal and frontal lobes, and is associated with high mortality and significant long-term neurological sequelae. Despite advances in antiviral therapy, HSE remains the leading cause of sporadic viral encephalitis worldwide.

The pathogenesis of HSE is complex and involves a dynamic interplay between viral neuro-invasion, host immune responses, and genetic predisposition [5]. Increasing evidence suggests that host intrinsic and innate immune responses within CNS-resident cells play a decisive role in limiting viral replication and preventing disease progression [5]. Conversely, defects in these pathways can lead to uncontrolled viral dissemination and severe neurological damage [6,7].

In this review, we summarise the current understanding of HSV and HSE, with a particular focus on epidemiology, antiviral immune responses, genetic susceptibility, and recent advances in therapeutic strategies. A comprehensive understanding of these aspects is essential for improving early diagnosis, optimizing treatment, and developing targeted interventions for HSE [8].

## 2. Epidemiology of HSV and HSE

HSV-1 and HSV-2 are widely distributed in the human population [2]. HSV-1 is primarily transmitted through oral contact and is commonly acquired during childhood, whereas HSV-2 is mainly transmitted through sexual contact and typically manifests later in life [3,4]. Following primary infection, both viruses establish latency in sensory neurons, allowing lifelong persistence and periodic reactivation [9].

HSE is a rare but severe complication of HSV infection, with an estimated incidence of approximately 2–4 cases per million individuals annually. HSV-1 accounts for over 90% of HSE cases in adults, while HSV-2 is more frequently associated with neonatal encephalitis [1,10]. The disease exhibits a bimodal age distribution, with higher incidence observed in infants and older adults.

A critical advance in understanding the long-term outcomes of HSE is the recognition that a subset of survivors develops secondary, antibody-mediated neurological syndromes. In a prospective nationwide cohort, approximately 27% of HSE survivors developed autoimmune encephalitis during follow-up visit [11]. Observations by Prüss et al. demonstrated that patients who experienced clinical relapses after HSE frequently harbour antibodies against the N-methyl-D-aspartate receptor (NMDAR), even though PCR for HSV is negative [12]. Nevertheless, post-HSE autoimmune encephalitis is not confined to anti-NMDAR cases; other neuronal surface antibodies and antibody-negative presentations have also been documented [11]. This syndrome typically emerges 2–6 weeks after the initiation of *acyclovir* therapy, presenting with new-onset or worsening neuropsychiatric symptoms, movement disorders, seizures, and autonomic instability—features that help distinguish it from viral recrudescence as well as from fixed neurological sequelae. Mechanistically, HSV-induced neuronal lysis is thought to expose neuronal surface antigens, triggering a robust adaptive immune response that is clinically manifested only after the acute infection has been controlled. The existence of this biphasic disease course has immediate clinical implications: deteriorating patients require prompt testing for a broad panel of neuronal surface antibodies in serum and cerebrospinal fluid, and if an autoimmune aetiology is confirmed, immunomodulatory treatment should be initiated without delay.

Despite the widespread prevalence of HSV infection, only a small proportion of infected individuals develop HSE, suggesting that host-specific factors play a critical role in disease susceptibility [13,14,15]. Mortality rates of untreated HSE can exceed 70%; however, early administration of intravenous acyclovir has reduced mortality to approximately 10–20% [1]. Nevertheless, a substantial proportion of survivors experience long-term neurological impairments, including cognitive dysfunction, seizures, and behavioral changes (Table 1) [7].

## 3. Immune Responses to HSV

### 3.1. Innate Immune Responses

**Figure 1:**
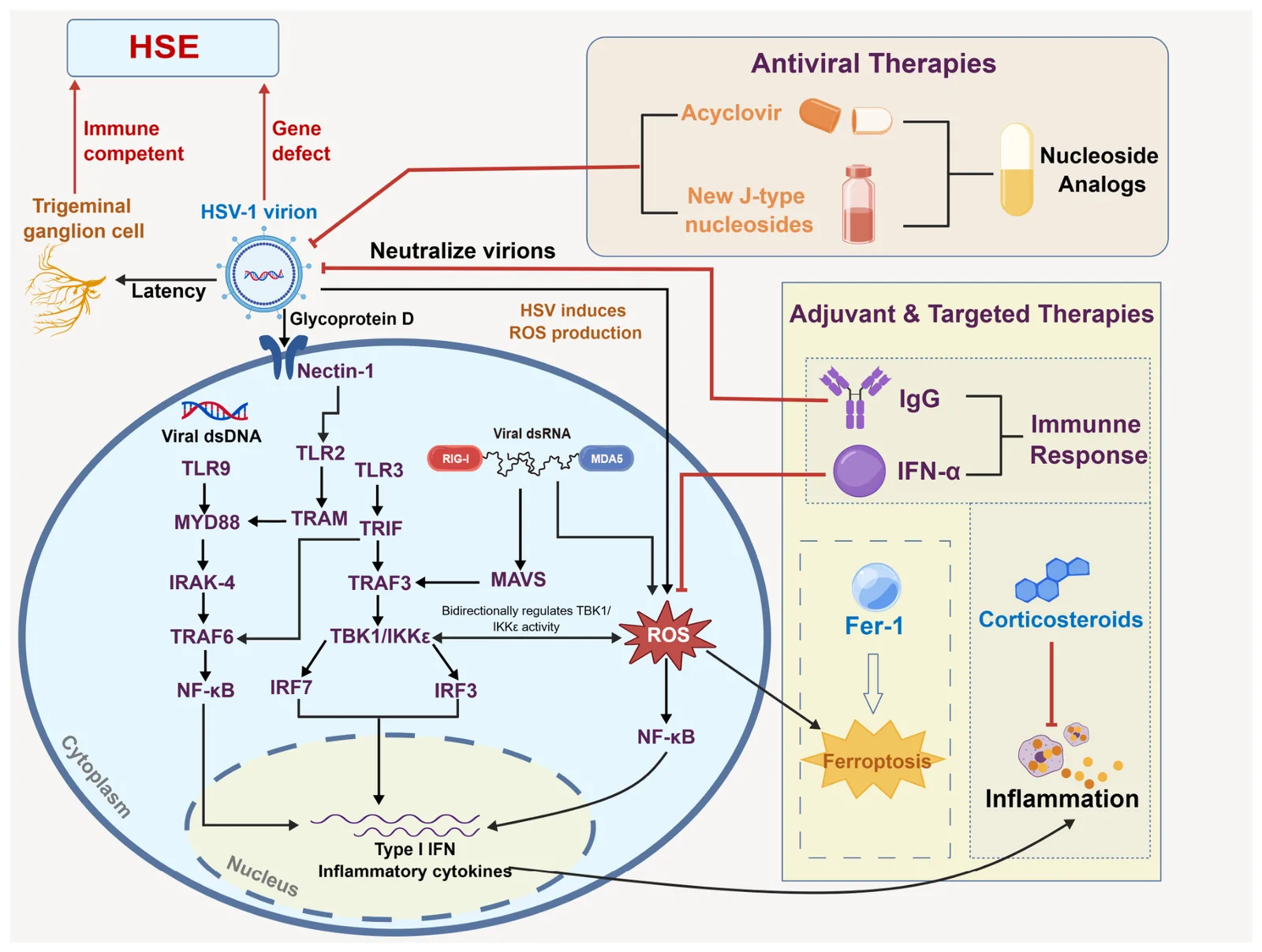
Schematic representation of innate immune recognition of HSV-1 and therapeutic strategies. Upon HSV-1 entry, the viral glycoprotein D (gD) binds the cellular receptor nectin-1, triggering the downstream fusion machinery (gB/gH/gL) to release the nucleocapsid. Host pattern recognition receptors (PRRs) sense viral components in distinct compartments: plasma membrane TLR2 and endosomal TLR9 signal via MyD88-IRAK-4-TRAF6 to activate NF-κB; endosomal TLR3 signals via TRIF to activate both the TRAF3-TBK1/IKKε-IRF3/IRF7 axis (inducing type I IFN) and NF-κB; and cytoplasmic RIG-I/MDA5 signal via MAVS-TRAF3-TBK1/IKKε to activate IRF3/IRF7. HSV-1 infection also triggers mitochondrial reactive oxygen species (ROS) production, promoting NF-κB activation and ferroptosis. Therapeutic interventions include (1) antiviral agents (acyclovir, J-type nucleosides); (2) adjuvant therapies (e.g., neutralizing IgG, type I IFNs); and (3) pathology-targeted strategies (Fer-1 for ferroptosis; corticosteroids for inflammation). Solid borders and filled arrows, established antivirals; dashed borders/open arrows, preclinical-stage therapies (e.g., ferroptosis inhibitors); dotted borders/shaded boxes, adjunctive strategies under clinical investigation (e.g., corticosteroids).

The innate immune system constitutes the first line of defense against HSV infection [17]. Upon viral entry, host cells detect HSV-derived nucleic acids through pattern recognition receptors (PRRs), including Toll-like receptors (TLRs), RIG-I-like receptors (RLRs), and cytosolic DNA sensors, such as cGAS (Figure 1) [18,19].

Among these pathways, the induction of type I IFNs is a central antiviral mechanism (Table 2) [20,21]. IFNs exert their effects by inducing the expression of IFN-stimulated genes (ISGs), which inhibit viral replication, enhance antigen presentation, and promote the activation of natural killer (NK) cells and T lymphocytes [22].

The TLR3/TRIF pathway plays a particularly important role in sensing double-stranded RNA intermediates generated during HSV replication. Activation of TLR3 leads to downstream signaling through TRIF, resulting in the activation of IRF3 and NF-κB and subsequent production of type I IFNs [18]. Similarly, the RIG-I/MAVS pathway detects cytosolic RNA species and contributes to antiviral signaling [28,29].

The cGAS/STING pathway is the primary sensor of HSV DNA in the cytoplasm. Upon recognition of viral DNA, cGAS synthesizes cyclic GMP-AMP (cGAMP), which activates STING and triggers downstream signaling cascades, leading to type I IFN production [19]. This pathway is particularly important in CNS-resident cells, including microglia and astrocytes [30,31]. Notably, activated STING signaling serves as an upstream trigger for NLRP3 inflammasome assembly, establishing critical cross-talk between nucleic acid sensing and pyroptotic inflammatory responses [32] (Figure 1).

Targeting the STING-TBK1-IRF3 signaling cascade represents a promising antiviral strategy. *Prunella vulgaris* polysaccharides have been shown to potentiate STING-dependent TBK1 phosphorylation and IRF3 nuclear translocation, augmenting IFNs production and restricting HSV-1 replication in cell culture systems [33]. Nevertheless, these observations remain limited to in vitro models and have not been validated in HSE-relevant CNS settings.

While these pathways are essential for antiviral defense, excessive activation can contribute to immunopathology. In the context of HSE, overproduction of pro-inflammatory cytokines and activation of inflammasomes can lead to neuronal damage and disruption of the blood–brain barrier (BBB) [5,13].

### 3.2. Adaptive Immune Responses

Adaptive immunity plays a critical role in controlling HSV infection and preventing reactivation [17]. CD8^+^ T cells recognize viral peptides presented by MHC class I molecules and eliminate infected cells through cytotoxic mechanisms, including the release of perforin and granzymes. They also produce IFN-γ, which further suppresses viral replication [1].

CD4^+^ T cells contribute by supporting CD8^+^ T cell responses and facilitating B cell activation. Th1 cells produce cytokines such as IFN-γ and IL-2, which enhance antiviral immunity [3]. Tissue-resident memory T cells (TRMs) in mucosal tissues provide rapid local immune responses upon viral reactivation [17,34].

B cells contribute to humoral immunity by producing neutralizing antibodies against viral glycoproteins, such as gB and gD, which block viral entry and dissemination. Although antibodies play a limited role in controlling latent infection, they are important in restricting viral spread during primary infection [17].

Dendritic cells serve as a critical link between innate and adaptive immunity by capturing viral antigens and presenting them to T cells [17,35]. However, HSV has evolved multiple mechanisms to evade immune detection, including inhibition of antigen presentation and interference with IFN signaling [29].

In conclusion, clinically, CD8^+^ T-cell dysfunction has been associated with HSV reactivation, whereas TRMs in sensory ganglia are thought to restrain viral recurrence.

## 4. Pathogenesis of HSE

HSE is a severe complication of HSV infection, primarily caused by HSV-1, but it has also been reported in neonatal HSV-2-induced CNS disease. HSE represents a significant proportion of sporadic viral encephalitis cases and can lead to life-threatening outcomes, particularly in neonates and individuals over 50 years of age [1,7]. Advances in antiviral therapy, such as the use of acyclovir, have significantly improved clinical outcomes for HSE patients; however, neurological sequelae remain a major concern for survivors (Table 3) [1,13].

**Figure 2:**
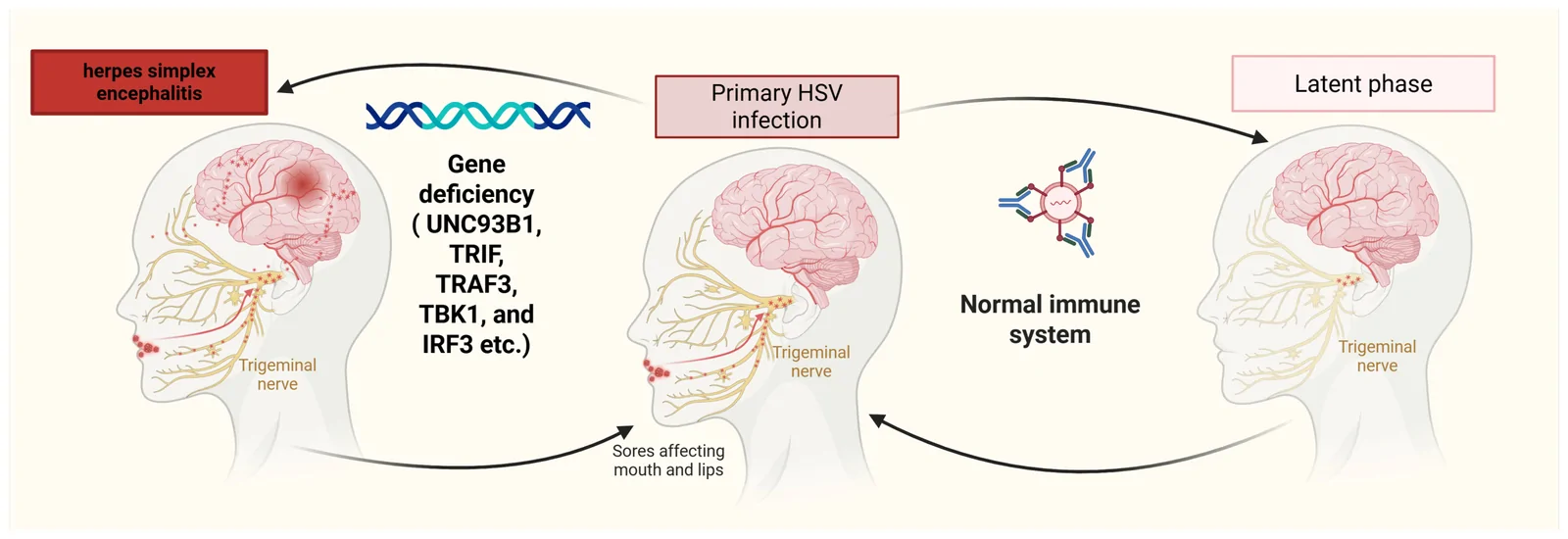
Host genetic susceptibility and antiviral immunity shape the pathogenesis and outcomes of HSE. This schematic illustrates how genetic defects in host antiviral immunity within the CNS predispose individuals to HSE. Recent advances have identified that mutations impairing the TLR3 pathway—including its chaperone UNC93B1, the adaptor TRIF, the signaling intermediate TRAF3, the kinase TBK1, and the transcription factor IRF3—may abrogate type I IFN production in CNS-resident cells upon HSV infection. These inborn errors of immunity underscore the indispensable role of intact TLR3–type I IFN signaling in brain-resident cells and provide a molecular explanation for the selective vulnerability to HSE observed in affected patients.

Recent research on HSE has focused on elucidating the immune responses involved in protecting the CNS from HSV infection, particularly the role of TLR3 and its pathways in recognizing and responding to viral invasion. Studies have emphasized the critical role of innate immune pattern recognition receptors, such as TLR3, in detecting viral components and inducing type I IFN expression to combat HSV infection. Furthermore, investigations into the molecular mechanisms underlying TLR3-mediated immune responses in CNS cells have provided valuable insights into susceptibility to HSV infection and identified potential therapeutic targets for enhancing antiviral immunity (Figure 2).

In conclusion, ongoing research efforts aimed at elucidating virus–host interactions, deciphering the immunological mechanisms underlying viral infections, developing novel therapeutic approaches, and advancing vaccine strategies are critical for improving clinical outcomes in HSE and reducing the burden of HSV-related complications. By leveraging insights into viral pathogenesis and host immune responses, we can develop more effective interventions to mitigate the impact of HSV infections on the CNS and enhance antiviral immunity.

### 4.1. Viral Entry and Neuro-Invasion

After HSV-1/2 infects the host through mucosa or damaged skin, heparan sulfate proteoglycans (HSPGs) bind to the surface receptors of epithelial cells (such as nectin-1 or HVEM), promoting the fusion of the viral envelope with the cell membrane [23]. Sensory nerve endings (such as trigeminal or sciatic nerve fibres) take up the virus and transport it to the neuronal cell body via retrograde axonal transport along the microtubule system. Latency-associated transcript (LAT) RNA inhibits expression of viral lytic genes and neuronal apoptosis, enabling the HSV genome to persist in sensory ganglia as an episomal circular DNA, thereby establishing latent infection [9,16]. Upon reactivation following external stimuli, the virus may spread to the CNS. Experimental studies in animal models suggest that anterograde axonal transport and trans-synaptic transmission represent potential routes of neuroinvasion [26,27]; however, the precise mechanism(s) by which HSV reaches the brain parenchyma in human HSE remain incompletely resolved. HSV infection promotes production of pro-inflammatory cytokines (such as TNF-α, IL-6) and matrix metalloproteinase-9 (MMP-9), which disrupt tight junctions of the BBB, facilitating viral entry into the brain parenchyma [5,13].

The invasion process of HSV follows a typical viral entry pattern and can be divided into three consecutive stages: initial attachment, membrane fusion, and stable pore formation. This process begins with the specific binding of viral envelope glycoprotein C (gC) to the sulfated heparan sulfate proteoglycans on the surface of the host cell, mediating the initial adsorption of the virus. Subsequently, viral envelope glycoprotein D (gD) binds with high affinity to the herpesvirus entry mediator (HVEM) on the surface of the host cell, achieving stable attachment of the virus. This dual receptor recognition mechanism prompts the close contact between the viral envelope and the host cell membrane, thereby activating the interaction between other envelope glycoproteins and cell surface molecules. It is worth noting that after gD binds to HVEM, it undergoes conformational changes, shifting from an auto-inhibitory closed state to an active state, and forms a functional unit with the viral glycoprotein H (gH)/L (gL) complex, inducing membrane semi-fusion, and subsequently activating gB. Glycoprotein B (gB) cooperates with the gH/gL complex to form stable fusion pores on the host cell membrane, mediating the intracellular release of the viral nucleocapsid, and during this process, gB also participates in the regulation of viral invasion through interactions with cell surface glycosaminoglycans [24,25].

### 4.2. Molecular Mechanisms of Neurotoxicity

#### 4.2.1. Neurotoxicity Mediated Directly by Viral Proteins

The ICP34.5 and UL37 proteins of HSV-1 are key factors for the pathogenicity of the virus. ICP34.5 mainly regulates translation and innate immunity, while UL37 dominates viral transport and apoptosis inhibition. Together, they prolong the survival of infected cells, promote viral transmission and latent neuroinfection. During infection, ICP34.5 targets protein phosphatase 1α (PP1α) to dephosphorylate eIF2α^,^ where eIF2 is a key factor for cap-dependent translation initiation, and the phosphorylation of the eIF2α subunit inhibits the formation of the 80S ribosomal complex by inhibiting guanine nucleotide exchange [40]. HSV antagonizes eIF2α phosphorylation through its pathogenic protein ICP34.5 to maintain viral protein synthesis, and ICP34.5 binds to TBK1 to inhibit IFN production and binds to Beclin-1 to inhibit Beclin-1-mediated autophagy [41]. UL37 promotes viral capsid transport by binding to dynamin [8] and inhibits BAK/BAX activation to block the mitochondrial apoptotic pathway. The UL37 tegument protein of HSV-1 deamidates RIG-I to avoid innate immune activation induced by dsRNA [42,43].

#### 4.2.2. Neuroinflammation and Immune-Mediated Injury

Neuroinflammation and immune-mediated damage in HSE are driven by virus-triggered innate immune responses. Microglia undergo pro-inflammatory activation and release cytokines such as IL-1β and TNF-α [5,30], while reactive astrocytes further amplify neuroinflammation. Adaptive immune components, including CD8^+^ cytotoxic T lymphocytes (via perforin-mediated cytotoxicity) and Th17 cells, together with the complement system, contribute to neuronal injury. Simultaneously, HSV-1 infection accelerates the degradation of nuclear factor E2-related factor 2 (Nrf2), a core transcription factor that governs antioxidant gene expression. This process disrupts cellular redox homeostasis and ultimately drives neuronal ferroptosis [6]. Upregulated MMP-9 and VEGF degrade tight junction proteins and increase vascular permeability, resulting in BBB disruption and further HSE progression [13].

## 5. Genetic Defects Related to HSE

Monogenic inborn errors of immunity are a major determinant of HSE susceptibility in children. The genetic defects and pathways discussed below, summarized in Table 4, have been characterized predominantly in the context of HSV-1 infection and are organized according to their characteristic clinical phenotypes and the strength of the associated evidence.

### 5.1. Isolated Childhood HSE with CNS-Restricted Susceptibility

Monogenic defects in the TLR3–type I IFN regulatory axis constitute the best-characterized genetic cause of isolated childhood HSE, in which encephalitis is the predominant or sole infectious manifestation, indicating CNS-restricted vulnerability [44]. TLR3 serves as an endosomal sensor for double-stranded RNA; autosomal recessive and dominant deficiencies impair TLR3-dependent induction of type I and type III IFNs, conferring high susceptibility to HSV-1 within the CNS [13,14,15,44]. UNC93B1 is required for intracellular trafficking of TLR3, TLR7, and TLR9; its deficiency abolishes TLR3 signaling and the downstream IFN response [44,45]. TRIF functions as the critical adaptor linking TLR3 and STING to IFN regulatory factor activation, and both recessive and dominant mutations disrupt dsRNA sensing and antiviral immunity [44,46]. TBK1 acts as an IFN-induced IRF3 kinase within the TLR3 pathway; autosomal dominant deficiency impairs IRF3 activation and is a well-documented cause of childhood HSE [44,47]. TRAF3 is essential for TLR3-dependent IFN induction, and dominant-negative mutations abolish the cellular response to TLR3 stimulation [15,44]. IRF3 haploinsufficiency (e.g., the R285Q variant) blocks S386 phosphorylation and dimerization, eliminating IFN induction via the TLR3–TRIF pathway in CNS-resident cells, particularly cortical neurons, thereby explaining the brain-restricted phenotype [44,58]. *GTF3A* encodes TFIIIA, which governs biogenesis of the endogenous RIG-I ligand *RNA5SP141*; pathogenic variants disrupt this cell-intrinsic antiviral axis and increase HSV-1 replication [48]. Collectively, these defects establish that intact TLR3- and RIG-I-dependent IFN signaling in the CNS is essential for protective immunity against primary HSV-1 infection in childhood [44].

### 5.2. Brainstem-Predominant Viral Encephalitis

Biallelic hypomorphic mutations in *DBR1* cause a distinct anatomical phenotype characterized by brainstem-predominant viral encephalitis, including HSV-1-associated brainstem HSE [49]. DBR1 is the only human RNA lariat debranching enzyme and exhibits preferential expression in the brainstem and spinal cord, accounting for the regional predilection. Loss-of-function variants trigger intracellular accumulation of RNA lariats, which interferes with stress-granule formation and downstream protein kinase R (PKR)-mediated antiviral immunity [50]. Importantly, TLR3 and type I IFN signaling remain intact in *DBR1* deficiency, establishing a TLR3–type I IFN-independent genetic risk factor with a neuroanatomical signature distinct from the forebrain-predominant HSE seen in TLR3-pathway defects [44].

### 5.3. Neuron-Intrinsic Forebrain HSE

Rare heterozygous variants in *SNORA31* predispose individuals to forebrain HSE through a neuron-autonomous mechanism [51]. *SNORA31* encodes an H/ACA box small nucleolar RNA that mediates ribosomal RNA pseudouridylation. Functional assays using patient-derived cortical neurons generated from induced pluripotent stem cells (iPSCs) confirm that *SNORA31* insufficiency selectively weakens the intrinsic neuronal antiviral program triggered by HSV-1, while basal TLR3–type I IFN signaling remains functional under extracellular stimulation; exogenous IFNs can rescue the viral susceptibility phenotype [51]. This neuron-intrinsic defect contrasts with DBR1-associated brainstem disease and demonstrates that forebrain HSE can arise from selective impairment of cell-intrinsic immunity without systemic IFN deficiency [44].

### 5.4. Broader Systemic Susceptibility to Viral Infection

In contrast to the CNS-restricted defects described above, mutations in *IKBKG* (encoding NEMO), *STAT1*, *STAT2*, *IFNAR1*, and *IFNAR2* confer widespread susceptibility to viral infections, and HSE represents only one, often rare, clinical manifestation within a broader syndromic phenotype. X-linked *IKBKG* deficiency impairs NF-κB activation downstream of TLR3 and multiple other innate pathways, resulting in defective IFN and cytokine production; HSV-1 encephalitis has been reported, typically in males, but affected individuals also suffer from bacterial and other viral infections [52]. Autosomal recessive or dominant *STAT1* deficiency disrupts both type I and type II IFN signaling and ISG expression, causing severe viral infections including HSE, although the clinical spectrum is broad [53,54]. Autosomal recessive *STAT2* deficiency abolishes type I IFN signaling via loss of ISGF3 activity; although in vitro studies confirm cellular susceptibility to HSV-1, clinical HSE is rare [44,55]. Complete autosomal recessive deficiency of *IFNAR1* or *IFNAR2* abrogates type I IFN responsiveness entirely, leading to life-threatening systemic viral infections; HSE has been documented in isolated case reports, but the predominant phenotype is broad viral susceptibility rather than isolated encephalitis [44,56,57]. For these genes, the link to HSE is supported by mechanistic plausibility and sporadic clinical observations, yet the evidence is less specific than that for TLR3-pathway defects or *SNORA31*.

## 6. Therapeutic Advances

### 6.1. Antiviral Therapies

At present, the management of both HSV-1- and HSV-2-related HSE mainly relies on pathogen-specific antiviral therapy. Intravenous *acyclovir* is the established first-line treatment for HSE. *Acyclovir* is a synthetic acyclic purine nucleoside analogue that is phosphorylated by viral thymidine kinase in infected cells to form *acyclovir triphosphate*, which inhibits viral DNA polymerase and terminates viral DNA chain elongation [59]. Compared with other antiviral agents, *acyclovir* is associated with fewer adverse reactions, exhibits low toxicity, and permits sustained administration, making it the standard therapy for HSE. In cases of *acyclovir* resistance or intolerance, alternative agents may be considered as salvage therapy; foscarnet, a pyrophosphate analogue, inhibits viral DNA polymerase directly without requiring phosphorylation by viral thymidine kinase, and *cidofovir*, an acyclic nucleoside phosphonate, also retains activity against HSV, yet both agents are reserved for selected circumstances, such as suspected or confirmed *acyclovir*-resistant infections, and are not equivalent first-line options for HSE. It is essential to distinguish the treatment of HSE from that of mucocutaneous HSV disease: oral nucleoside analogues—including *valacyclovir*, a prodrug of *acyclovir* with higher oral bioavailability that achieves blood concentrations approximately five times those of oral *acyclovir*, as well as *famciclovir* and *penciclovir*—are effective for mucocutaneous HSV infections. However, these oral agents are not appropriate for acute HSE, which requires intravenous administration to achieve adequate drug concentrations in the CNS promptly. *Ganciclovir* and *valganciclovir* are primarily indicated for cytomegalovirus infections and should not be regarded as routine therapies for HSV infection or HSE (Table 5). Nucleoside analogues have significant limitations in the treatment of HSV-1 infection, including the generation of side effects, the emergence of drug-resistant strains, and limited duration of efficacy [60]. Therefore, new antiviral agents with distinct mechanisms of action are needed. Recent studies have shown that Janus-type nucleosides, which possess two different faces within a single molecule to mimic natural purine and pyrimidine systems, can induce lethal mutations in viruses by pairing with different natural bases through rotation around the glycosidic bond and are expected to become novel targeted antiviral drugs for HSV [60]. In addition, baicalein, a natural derivative compound, can inhibit the replication of HSV-1 and HSV-2 (an *acyclovir*-resistant strain) in vitro and represents a promising candidate for HSV therapy [61].

While nucleoside antivirals restrain productive HSV replication yet cannot erase trigeminal latent viral reservoirs, CRISPR-Cas9 editing is under preclinical investigation as a potential strategy to target latent HSV-1. AAV-delivered CRISPR targeting HSV-1 ICP0 and ICP27 suppresses latent viral rebound in human brain organoid latency models, which lack the full immunological and anatomical complexity of the CNS in vivo [62]. In a latent rabbit keratitis model, intravenous administration of a single AAV9 vector encoding SaCas9 paired with dual gRNAs (g2g1) reduced HSV-1 DNA by 50% and LAT RNA by 60% to 80% within trigeminal ganglia [63]; however, keratitis models do not recapitulate the pathophysiology of HSE, and these findings cannot be directly extrapolated to CNS infection. The HELP VLP-mRNA platform avoids genomic integration via VSV-G-dependent retrograde neural trafficking to trigeminal ganglia and minimizes off-target effects [64], though it has only been evaluated in preclinical neural specimens and murine keratitis models, with no validation in HSE models or human CNS disease [65]. AAV sustains editing activity but risks insertion mutagenesis, whereas HELP offers improved safety and neural tropism; whether either approach could ultimately be translated to prevent or treat HSE remains speculative and will require dedicated CNS-targeted preclinical and clinical evaluation. Moreover, because nucleoside analogues only suppress lytic replication and do not eliminate latent HSV in trigeminal ganglia, HSE can still occur despite adequate antiviral treatment, and the risk of recurrence persists throughout the patient’s lifetime, especially under immunocompromising conditions.

**Table 5:** Therapeutic Strategies for HSV Infection and HSE.

Therapeutic Class/Strategy	Agent/Example	Mechanism of Action	Advantages/Limitations	References
First-line therapy for acute HSE	Acyclovir (intravenous infusion)	Viral TK-mediated phosphorylation → DNA chain termination	Established first-line treatment for acute HSE; low toxicity; sustained administration	[59,60]
Salvage therapy for acyclovir-resistant or -intolerant HSE	Foscarnet, cidofovir	Foscarnet: pyrophosphate analogue; inhibits viral DNA polymerase directly without TK phosphorylation. Cidofovir: acyclic nucleoside phosphonate	Reserved for selected circumstances (e.g., confirmed acyclovir resistance); not equivalent first-line options	[59,60]
Oral nucleoside analogues for mucocutaneous HSV	Valacyclovir, famciclovir, penciclovir	Viral TK-mediated phosphorylation → DNA chain termination; valacyclovir is an oral acyclovir prodrug with ~5-fold higher bioavailability; famciclovir is an oral penciclovir prodrug	Effective for mucocutaneous HSV infection; not appropriate for acute HSE	[59,60]
Janus-type nucleosides	–	Ambiguous base-pairing → lethal mutagenesis	Investigational; may overcome resistance	[60]
Natural flavonoid	Baicalein	Inhibits replication of wild-type and ACV-resistant HSV-1	In vitro evidence only; potential adjunct	[61]
Ferroptosis inhibitors	Ferrostatin-1, Liproxstatin-1	Block oxidative stress-induced ferroptosis	Preclinical; protect against HSV-induced cell death	[6]
Immunomodulators	IFN-α, Intravenous immunoglobulin (IVIG)	Enhance host antiviral immune response	Adjunctive approach; no consistent evidence from controlled trials supporting efficacy against post-HSE sequelae	[66,67]
Corticosteroids	Dexamethasone	Attenuate neuroinflammation and cerebral oedema	Phase 3 trial (DexEnceph) showed no significant improvement in verbal memory at 26 weeks versus aciclovir alone; satisfactory safety profile; role in acute HSE remains uncertain	[68]
Precision TLR/RLR agonists	Targeting TLR3-TRIF, RIG-I/MDA-5	Enhance type I IFN and innate antiviral responses	Potential therapy for patients with genetic IFN defects	[13,44,69]
ROS/Nrf2 modulators	Antioxidants, Nrf2 activators	Restore redox balance; inhibit ferroptosis	Emerging strategy based on ROS-mediated innate signaling	[6,70]

### 6.2. Adjunctive Therapies

Upon HSV-1/2 infection, the virus establishes lifelong latency in sensory ganglia and cannot be eradicated by current antiviral therapy, predisposing to recurrent mucocutaneous episodes. Antiviral drugs such as acyclovir and valacyclovir suppress acute viral replication but do not eliminate the latent reservoir. Adjunctive strategies in HSE aim to attenuate pathological host immune responses and mitigate neurological injury. Ferrostatin-1 (Fer-1) and liproxstatin-1 have been shown to inhibit HSV-induced ferroptotic cell death [6].

The role of adjunctive corticosteroids in acute HSE remains an area of ongoing clinical uncertainty. The phase 3 DexEnceph trial, a multicentre, observer-blind, randomised controlled trial conducted at 53 hospitals in the UK, compared adjunctive intravenous dexamethasone (10 mg four times daily for 4 days) plus standard aciclovir with aciclovir alone in 94 adults with PCR-confirmed HSE [68]. The primary outcome—verbal memory score at 26 weeks, assessed by the Wechsler Memory Scale-IV auditory memory index—did not differ significantly between groups (adjusted mean difference 1·77, 95% CI −9·57 to 13·12; *p* = 0·76). The trial demonstrated a satisfactory safety profile, with no evidence of increased viral replication in the dexamethasone group. Although an exploratory post hoc analysis suggested that earlier corticosteroid administration might be associated with better outcomes, the study did not establish a general clinical benefit for routine adjunctive dexamethasone in HSE. Consequently, routine adjunctive dexamethasone cannot be recommended for all adult HSE patients. Any use should be individualized (e.g., severe cerebral oedema, very early presentation) and accompanied by close safety monitoring. Although the trial did not show increased viral replication, this does not obviate the need for caution.

Following the initial illness, patients may exhibit one of three distinct clinical trajectories: fixed neurological sequelae attributable to parenchymal injury, recurrent viral HSE, or secondary autoimmune encephalitis [13]. The latter must be distinguished from both viral relapse and irreversible sequelae, since management differs fundamentally. Evidence from broader autoimmune encephalitis cohorts, including the Titulaer anti-NMDAR encephalitis study, suggests that early immunotherapy improves long-term functional outcomes [71]; however, direct prospective evidence specific to post-HSE autoimmune encephalitis remains limited, and current clinical recommendations draw heavily on observational data from post-HSE cohorts. The efficacy of type I IFNs and intravenous immunoglobulins in ameliorating post-HSE symptoms has not been established in controlled studies, and these agents should not be regarded as generic therapy for undefined post-HSE complaints without clear diagnostic categorisation.

### 6.3. Emerging and Experimental Therapeutic Approaches

The synergistic interplay between TLR and RLR pathways facilitate rapid and robust immune responses following HSV-1 infection, underscoring these receptors as potential therapeutic targets for enhancing HSE treatment [18,44,46]. Other therapeutic strategies including intrathecal IFN-α (0.5–1.2 × 10^6^ IU/day) [72], which have improved outcomes have been observed in isolated refractory HSE case reports, yet controlled phase II trial data are absent. STING agonist DMXAA reduces HSV-1 replication in vitro and in vivo yet is most effective prophylactically and species-restricted; human-active agonists (e.g., 2′3′-cGAMP) are in development [73,74]. CRISPR-Cas9 targeting ICP0/ICP27 eliminated HSV-1 shedding in 92% of rabbit keratitis eyes and reduced ganglionic viral DNA/RNA, though CNS delivery and off-target effects persist [63]. Collectively, these immunomodulatory, STING-activating, and gene-editing approaches move beyond antiviral suppression toward eradication, but each face distinct translational hurdles requiring further investigation.

The clinical approach to post-HSE autoimmune encephalitis could also be consolidated according to the research update. When a patient with prior HSE presents with new neurological symptoms, distinguishing autoimmune relapse from viral reactivation is the first critical step. Cerebrospinal fluid (CSF) PCR for HSV-1 DNA and concomitant serum/CSF neuronal-surface antibody panels are essential, as a positive result of PCR suggests active viral replication, whereas antibody positivity indicates an autoimmune process, often with characteristic MRI changes (e.g., mesial temporal or extratemporal involvement). Both serum and CSF should be tested for neuronal-surface antibodies, because seronegative CSF can still harbor diagnostic antibodies and vice versa. Once autoimmune aetiology is confirmed and active viral replication has been rigorously excluded, immunomodulatory therapy (corticosteroids, IVIG, or plasma exchange) should be initiated without delay. Early recognition and prompt immune intervention are associated with better functional outcomes, whereas unnecessary prolongation of antiviral therapy alone may delay effective treatment and worsen prognosis.

## 7. Discussion

While the causal link between immune signaling pathway defects and HSE is well-established, this framework has profound gaps that need critical consideration. A primary unresolved issue is the discordance between human genetics and animal models. Human HSE predominantly maps to TLR3 and its adaptors, yet murine data highlight cytosolic sensors, such as RIG-I and cGAS, in neuronal antiviral defense, which is rarely implicated in human disease [44]. This raises a crucial, unanswered question: do mouse knockouts merely represent artificial systems, or do they reflect compensatory mechanisms in humans? A critical translational gap lies in the species- and cell-type-specific innate immune wiring between humans and mice. Human cortical neurons predominantly engage TLR3-dependent type I IFN signaling upon HSV-1 exposure, whereas mouse microglia primarily rely on the cGAS–STING and RIG-I pathways. This dichotomy is not merely a matter of receptor preference but reflects fundamental differences in the cellular sources of IFN responses across species. Consequently, findings from murine HSE models, particularly those involving neuronal intrinsic immunity, cannot be directly extrapolated to the human CNS environment. Such caution is essential when interpreting preclinical data, as the murine immune architecture may either overestimate or obscure pathways that are truly operative in human HSE pathogenesis [75,76].

Furthermore, the cellular autonomy of these defects deserves to be assessed. Neurons are the primary targets of HSV-1, yet most functional assays rely on patient fibroblasts or peripheral blood mononuclear cells [51]. Human iPSC-derived CNS models underscore that fibroblasts frequently fail to recapitulate neuron-specific antiviral phenotypes, reinforcing the limitation of peripheral cell readouts for HSE-associated variants [77]. We critically lack an understanding of whether mutations in DBR1 or SNORA31 predispose neurons to death via distinct RNA-metabolism pathways, thereby questioning the universal applicability of a purely immunological model [49,50]. Additionally, functional validation of novel human immune gene variants frequently uses overexpression systems that lack physiological relevance, overlooking cell-type-specific landscapes across neurons, astrocytes and microglia of the human CNS [78].

Clinically, the therapeutic implementation of immunomodulation in HSE remains unresolved. While enhancing IFN responses appears logical given the essential role of TLR3-dependent type I IFN immunity in the CNS [69], systemic immunomodulatory agents carry unquantified risks. Ruxolitinib, a JAK1/2 inhibitor that suppresses type I IFN signaling, can promote herpesvirus reactivation and may exacerbate CNS inflammation or viremia if administered outside a narrow window [8]. Heterogeneous blood–brain barrier penetration further complicates risk–benefit evaluation for its repurposing in HSE therapy [79,80]. Therefore, despite considerable enthusiasm surrounding several preclinical candidates, their translation into clinical practice for HSE remains fraught with unaddressed obstacles. Agents such as Janus-type nucleosides, baicalein, ferroptosis inhibitors, STING agonists, and CRISPR-Cas9 targeting latent HSV-1 have all shown activity in vitro or in animal models, yet none have been tested in controlled human HSE trials or demonstrated any clinical efficacy in patients. For CRISPR-based approaches in particular, three major barriers preclude immediate clinical application: inefficient delivery across the BBB to sensory ganglia, the potential for off-target genomic edits in neural tissues, and the lack of animal models that faithfully recapitulate the human latent-reactivation cycle and HSE immunopathology. Moreover, even if delivery and safety challenges were overcome, the functional consequences of editing latent viral genomes in non-dividing neurons remain poorly understood. Therefore, all these strategies must be regarded as strictly investigational, and their current mention in the literature should not be misinterpreted as readiness for clinical use.

## 8. Conclusions

In conclusion, HSE remains a formidable clinical challenge, with mortality and long-term morbidity remaining unacceptably high despite the availability of acyclovir. The striking disparity between the high seroprevalence of HSV-1 and the rare occurrence of HSE underscores that host genetic determinants, rather than viral factors alone, contribute to disease susceptibility. Over the past decade, the identification of monogenic inborn errors of immunity has advanced our understanding of HSE, revealing that cell-intrinsic antiviral responses in the CNS are important for controlling viral replication. Defects in molecules, such as, UNC93B1, TRIF, TRAF3, TBK1, IRF3, as well as *IFNAR1/2* and *STAT1/2*, compromise the production or signaling of type I IFNs, thereby permitting unchecked HSV proliferation in brain-resident cells. These insights provide a molecular basis for susceptibility in affected individuals. However, the genetic defects discussed are rare, exhibit incomplete penetrance and age-dependent expression, and show heterogeneous clinical relevance. Consequently, they do not support population-level risk prediction. Moving forward, genetic evaluation may offer potential value in selected patients, particularly children with otherwise unexplained HSE, to inform clinical assessment and potentially guide management in individual cases, though genotype-directed therapy remains investigational. Importantly, the majority of HSE patients do not harbor any of the known monogenic mutations. Genetic testing is therefore indicated only for selected cases, particularly unexplained pediatric HSE or those with a suggestive family history; it should not be used for population-based risk screening. Moreover, further research is needed to elucidate the contributions of other immune pathways, the role of glial cells, and the potential for gene therapy or cytokine supplementation. Ultimately, bridging basic immunology with clinical practice will be essential to reduce the devastating burden of HSE and improve patient outcomes.

## Figures and Tables

**Table 1:** Epidemiology and Clinical Features of HSV Infection and HSE.

Feature	HSV-1	HSV-2	Herpes Simplex Encephalitis (HSE)	References
Global seroprevalence	~67%	~13%	Incidence 2–4 per million/year	[1,2]
Primary transmission route	Oral (commonly childhood)	Sexual (later in life)	>90% adult cases caused by HSV-1	[1,3,4]
Latency site	Sensory neurons	Sensory neurons	Sensory neurons	[9,16]
Typical clinical presentation	Orolabial lesions, often asymptomatic	Genital lesions, often asymptomatic	Acute temporal/frontal lobe inflammation	[1,7]
Mortality	–	–	Untreated > 70%	[1]
Long-term sequelae	–	–	Cognitive impairment, seizures, behavioral changes	[7]

**Table 2:** Key Viral and Host Molecules in HSV Entry and Neuro-invasion.

Step	Viral Factor	Host Target/Receptor	Function & Mechanism	References
Initial attachment	gC	Heparan sulfate proteoglycans (HSPGs)	Mediates initial low-affinity adsorption to cell surface	[23]
Stable binding	gD	Herpesvirus entry mediator (HVEM), nectin-1	High-affinity binding; triggers conformational change, activates fusion	[23]
Membrane hemifusion	gH/gL complex	Host membrane	Forms functional unit with gD; induces hemifusion	[24,25]
Stable fusion pore	gB	Glycosaminoglycans, membrane	Cooperates with gH/gL; releases nucleocapsid into cytoplasm	[24,25]
Retrograde axonal transport	Capsid	Dynein/dynactin (via UL37-dynamin)	Transport to neuronal soma; establishes latency	[26,27]
Reactivation & CNS entry	–	Tight junctions	TNF-α, MMP-9 upregulation disrupts blood–brain barrier (BBB)	[5,13]

**Table 3:** Innate Immune Pathways in Anti-HSV Defense and HSE Pathogenesis.

Pathway/Sensor	Key Adaptor(s)	Primary Effector(s)	Protective Role in CNS	Molecular Pathology of HSE	References
TLR3	TRIF → IRF3, NF-κB	Type I IFNs, ISGs	Essential for CNS-intrinsic control of HSV-1; limits spread	Defects lead to HSE; no direct overactivation described	[15,18]
RIG-I/MDA-5	MAVS (IPS-1)	Type I IFNs	Cytosolic RNA sensing; synergistic with TLR3 pathway	UL37 deamidates RIG-I; impaired signaling enhances replication	[13,17,28]
cGAS/STING	STING → IRF3	Type I IFNs	DNA sensing in microglia/astrocytes	Activates NLRP3 inflammasome → IL-1β, neuroinflammation	[17,19,30]
TLR2/9-MyD88	MyD88 → NF-κB	Pro-inflammatory cytokines	Contributes to early cytokine milieu	Drives neuroinflammation, immune-mediated injury	[36,37,38]
NLRP3 inflammasome	ASC, caspase-1	Mature IL-1β, neutrophil recruitment	–	Amplifies inflammation, disrupts BBB	[5,13]
Nrf2 (antioxidant)	Keap1-Nrf2	Antioxidant genes	Maintains redox homeostasis; inhibits ferroptosis	HSV-1 degrades Nrf2 → oxidative stress, ferroptosis	[6,39]

**Table 4:** Monogenic Defects Associated with Susceptibility to HSE.

Gene	Encoded Protein	Inheritance	Impact on Innate Immune Pathway	Defect Consequence on Anti-HSV Immunity	References
*TLR3*	TLR3	AR, AD	Impaired dsRNA sensing in endosomes	Loss of IFN induction; high susceptibility to CNS HSV-1	[44]
*UNC93B1*	UNC93B1	AR	Defective intracellular trafficking of TLR3/7/9	Impaired TLR3 signaling → abolished IFN response	[44,45]
*TICAM1*	TRIF (TICAM1)	AR, AD	Disrupted TLR3- and STING-dependent signaling	Impaired IFN induction; defective antiviral response	[44,46]
*TBK1*	TBK1	AD	Defective kinase activity for IRF3 in TLR3 pathway	Impaired IFN-induced IRF3 activation; childhood HSE	[44,47]
*TRAF3*	TRAF3	AD	Impaired TLR3-dependent IFN induction	Lack of response to TLR3 stimulation; HSE predisposition	[15,44]
*IRF3*	IRF3	AD (haploinsufficiency)	R285Q mutation blocks S386 phosphorylation, no dimerization	Abolished IFN response to HSV-1 via TLR3-TRIF; HSE in adolescents	[44]
*GTF3A*	TFIIIA	–	Reduced transcription of RIG-I agonist RNAs (RNA polymerase III)	Impaired intracellular innate response; increased viral replication	[48]
*DBR1*	DBR1 (RNA lariat debranching enzyme)	AR	Disrupted RNA lariat turnover; impaired stress granule/PKR immunity; intact TLR3-type I IFN signaling	Increased susceptibility to brainstem HSV-1 encephalitis	[44,49,50]
*SNORA31 (non-coding RNA)*		AD	Impaired rRNA pseudouridylation; neuron-intrinsic antiviral dysfunction; intact basal TLR3-type I IFN signaling	Selective impairment of neuronal anti-HSV-1 immunity; susceptibility to forebrain HSE	[44,51]
*IKBKG*	NEMO (IKKγ)	X-linked	Defective NF-κB activation downstream of TLR3 and other innate pathways; impaired IFN and cytokine production	HSV-1 encephalitis (typically in males)	[52]
*STAT1*	STAT1	AR, AD	Impaired type I and type II IFN signaling; defective ISG expression	Susceptibility to severe viral infections including HSE	[53,54]
*STAT2*	STAT2	AR	Defective type I IFN signaling; loss of ISGF3 activity	In vitro susceptibility to HSV-1; predisposition to severe viral infections, rare clinical HSE	[44,55]
*IFNAR1*	IFNAR1	AR	Complete loss of type I IFN responsiveness	Severe viral infections, HSE	[56]
*IFNAR2*	IFNAR2	AR	Complete loss of type I IFN responsiveness	Abolished type I IFN-mediated antiviral activity; cellular susceptibility to HSV-1, rare HSE in clinical reports	[44,57]

## Data Availability

Data sharing is not applicable. No new data were created or analyzed in this study.
